# Biomedical features of flaxseed against different pathologic situations: A narrative review

**DOI:** 10.22038/ijbms.2021.49821.11378

**Published:** 2021-05

**Authors:** Babak Ebrahimi, Zohreh Nazmara, Negar Hassanzadeh, Atousa Yarahmadi, Neda Ghaffari, Fatemeh Hassani, Amirreza Liaghat, Leila Noori, Gholamreza Hassanzadeh

**Affiliations:** 1 Department of Anatomy, School of Medicine, Tehran University of Medical Sciences, Tehran, Iran; 2 Legal Medicine Research Center, Legal Medicine Organization, Tehran, Iran; 3 Department of Cardiology, Tehran Heart Center, Tehran University of Medical Sciences, Tehran, Iran; 4 Department of Embryology, Reproductive Biomedicine Research Center, Royan Institute for Reproductive Biomedicine, ACECR, Tehran, Iran; 5 Legal Medicine Research Center, Legal Medicine Organization, Mashhad, Iran; 6 Department of Anatomical Sciences, School of Medicine, Babol University of Medical Sciences, Babol, Iran; 7 Department of Neuroscience and Addiction Studies, School of Advanced Technologies in Medicine, Tehran University of Medical Sciences, Tehran, Iran

**Keywords:** Anticancer, Antidiabetic, Anti-oxidant, Biomedical, Flaxseed, Medicinal properties

## Abstract

Flaxseed is a plant that grows and is cultivated in more than 50 countries; the main flax producer countries are Canada, China, the United States, and India. The purpose of the present study was to overview the source, chemical compounds, and mechanisms of the therapeutic effects of this valuable plant. For writing this manuscript, we made a list of relevant keywords and phrases, and then we started searching for studies in PubMed, Scopus, and Web of Science databases. The main constituents of flaxseed include lipids, proteins, lignans, fibers, and minerals. Flaxseed is full of antioxidants such as tocopherols, betacarotene, cysteine, and methionine which result in a decrease in blood pressure, heart disease, hepatic and neurological disorders, and increased insulin sensitivity. Flaxseed is commonly used for its antidiabetic and anticancer activities and also it is beneficial for cardiovascular, gastrointestinal, hepatic, urological, and reproductive disorders, and because of these beneficial effects, it is recognized as a medical plant.

## Introduction

Flaxseed (*Linum usitatissimum*) has been planted since the initiation of civilization. *Linum usitatissimum *is the Latin name of flaxseed, which means very beneficial. At first, it emerged in the United States and was used for clothing fiber production (1, 2). Flaxseed is important in the food chain throughout the world because of its physiological advantages in preventing or treatment of diseases as a functional food. Flaxseed has been regarded as a Generally Recognized As Safe (GRAS) source of vitamins, minerals, proteins, and peptides (including bioactive cyclic peptides), lipids (including omega-3 and omega-6 polyunsaturated fatty acids), carbohydrates, lignans, and dietary fiber. Due to its beneficial features for health, it has been applied in specific diets. Due to a 12% growth in the annual rate of the flaxseed global marketing, it can be predicted that its worth may be increased to US$ 1.95 million (3). Many causes lead to the increasing popularity of flaxseed, such as alleviation of cardiovascular diseases, decreasing the risk of cancer, especially breast and prostate cancer, anti-inflammatory effects, lenient impressions, and reduction of menopause signs and osteoporosis (1). 

For writing this manuscript, we made a list of relevant keywords and phrases, and then we started searching studies in PubMed, Scopus, and Web of Science dases. This review is an attempt to demonstrate the different biological activities and medical properties of flaxseed as a traditional plant and its components in animal studies and some human trials. Particularly, we focused on the mechanisms underlying flaxseed effects on hepatic, neurological, cardiovascular, gastrointestinal, reproductive, and urological disorders, and also its anti-oxidant, antidiabetic, and anticancer properties.

The keywords that we used were: Flaxseed, anti-oxidant, antidiabetic, anticancer, biomedical, and medicinal properties. The study was conducted between March and June 2020. All the literature found in databases was included but there was a preference towards recent studies.

***Source and chemical compounds***

Nowadays, flaxseed is planted in more than 50 countries; the main flax producer countries are Canada, China, the United States, and India (4). Flaxseed is found in two types (1) brown and (2) yellow or golden. Brown and yellow flaxseeds have equal numbers of short-chain ω-3 fatty acids and identical nutritional features. A type of flaxseed called solin has a different oil profile and ω-3 fatty acids numbers compared with others. Brown flax is well-known for its usage in paints, varnish, fiber, and livestock feed. Flaxseed has multiple compounds with bioactive plant materials such as oil, protein, lignans, dietary fibers, vitamins (A, C, F, and E), and minerals (P, Mg, K, Na, Fe, Cu, Mn, and Zn) that impact several disorders that are summarized in Table 1 (5).

***Flaxseed oil/lipids***

**Figure 1:**
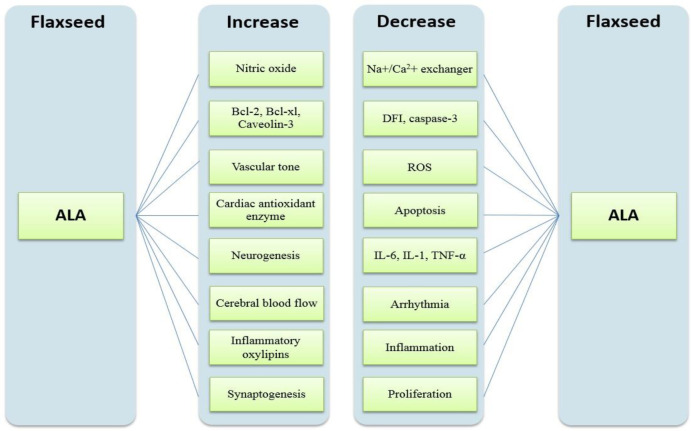
Impacts of α- linolenic acid (ALA) on different parts of the body

Flaxseed is the richest plant source of the ω-3 fatty acid, i.e., *α- *linolenic acid (ALA). Flaxseed oil is composed of 73% polyunsaturated fatty acids, 18% monosaturated fatty acids, and 9% saturated fatty acids (6, 7). Flaxseed oil is full of anti-oxidants such as *tocopherols* and *betacarotene*, nevertheless, common pure flaxseed oil oxidizes quickly after being exploited. The impacts of ALA on different parts of the body are summarized in Figure 1 (6).

***Proteins***

Flaxseed is built of 20%–30% protein, including almost 80% globulins (linin and conlinin) and 20% glutelin. The nutritional value and amino acid profile of flaxseed and soya are comparable. Flaxseed proteins consist of arginine, aspartic acid, glutamic acid, and a little lysine (8). The risk of cancer is decreased by flaxseed due to its high anti-oxidant levels such as cysteine and methionine. It also alleviates the risk of cardiovascular disease by peptides with bioactivities (9).

***Lignans***

**Figure 2:**
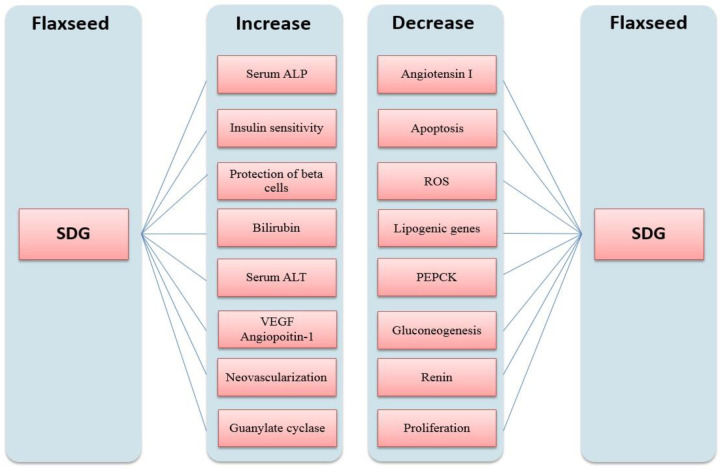
Effects of secoisolariciresinol diglucoside (SDG) on different parts of the body

Two cinnamic acid connections constitute *phenolic* compounds called plant lignans. Lignans are found everywhere in approximately all plants (10). Lignans are considered as both anti-oxidants and phytoestrogens. Low estrogen activity is exhibited by phytoestrogens in animals and humans. The quantity of lignans in flax is up to 800 times greater than other plants. Mainly secoisolariciresinol diglucoside (SDG) (294–700 mg/100 g), matairesinol (0.55 mg/100 g), lariciresinol (3.04 mg/100 g) and pinoresinol (3.32mg/100 g) construct flaxseed lignans. Ferulic acid (10.9 mg/g), chlorogenic acid (7.5 mg/g), gallic acid (2.8 mg/g) are the most common phenolic acids in defatted flaxseed. Also, other phenolic acids including p-coumaric acid glucosides, hydroxycinnamic acid glucosides, and 4-hydroxybenzoic acid are found to a lesser extent. Bacteria in the gut transform the SDG of flax to the lignans-enterodiol and enterolactone which cause useful outcomes such as estrogenic or anti-estrogenic and anti-oxidant effects (11). The effects of flax lignans in decreasing the progress of malignant tumors particularly hormone-sensitive ones such as breast, endometrium, and prostate cancers are encouraging. The effects of SDG on different parts of the body are summarized in Figure 2 (11).

***Dietary fiber***

**Figure 3:**
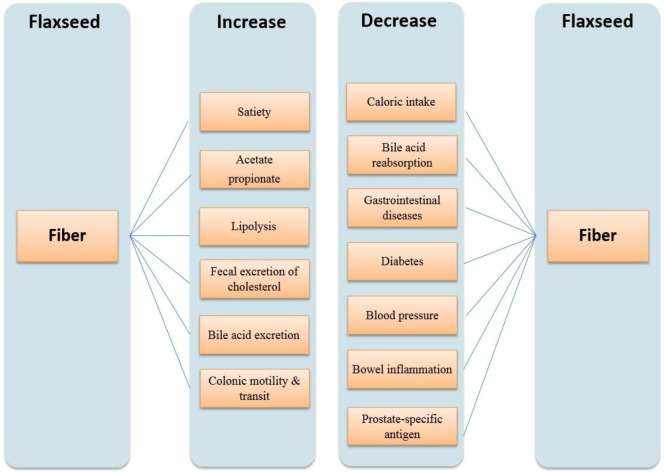
Effects of fibers on various parts of the body

Flaxseeds contain both solvable and unsolvable dietary fiber. The glaze of the outer layers of flaxseed turns it into an unrivaled oilseed (8). High advantages and potential functional features of flaxseed mucilage have made so valued. Flaxseed is constructed of 35–45% fiber: two-thirds unsolvable and one-third solvable fiber which comprises cellulose, hemicellulose, and lignin. It seems the mucilage of the seed cover is mainly made of soluble fiber. Mainly water-soluble polysaccharides constitute mucilaginous substances in flaxseed. Utilization conditions affect the recovery and purity of soluble fiber. The effects of fibers on various parts of the body are summarized in Figure 3 (12).

***Minerals***

Flaxseed is considered a rich source of minerals particularly phosphorous (650 mg/100 g), magnesium (350–431 mg/100 g), calcium (236–250 mg/100 g), and a low deal of sodium (27 mg/100g). Flaxseed comprises a great deal of potassium 5600–9200 mg/kg and high consumption of potassium reduces the risk of blood platelet aggregation, free radicals in the blood, and stroke. A small water-soluble and fat-soluble vitamin content can be found in flaxseed.

The quantity of *γ- tocopherol* (vitamin E) reaches up to 39.5 mg/100 g. γ- tocopherol not only acts as an anti-oxidant but also increases sodium expulsion in urine, which results in a decrease in blood pressure, heart disease, and Alzheimer’s risks (13).

***Anti-nutritional factors***

Anti-nutrients in flaxseed may create negative effects on human health. The main anti-nutrients are cyanogenic glycosides (CG), including linustatin (213–352 mg/100 g), neolinustatin (91–203 mg/100 g), and linmarin (32 mg/100 g). Phytic acid is another anti-nutrient in flaxseed ranging from 23 to 33 g/kg (14). The absorption of calcium, zinc, magnesium, copper, and iron is restricted by phytic acid. It should be noticed that the risk of poisoning with foodstuffs including CG is alleviated by sufficient processing. For example, more than 85 % of linustatin and neolinustatin were detoxified due to the heating of flaxseed for more than 2 hr at 200 °C (15).

***Anti-oxidant capacity of flaxseed***

Homeostasis is normally protected from reactive oxygen species via an endogenic anti-oxidant defense procedure but our body is constantly susceptible to oxidative attack which leads to oxidative stress (16). Typical anti-oxidant classification contains synthetic and natural s and also another method categorizes anti-oxidants as primary (chain-breaking) anti-oxidants or secondary (preventive) anti-oxidants. Primary anti-oxidants stabilize the radicals directly through mechanisms like donation of a hydrogen atom or single electron transfer, but secondary anti-oxidants inhibit the oxidant-producing pathways (17). It has been demonstrated that flaxseed contains high amounts of natural anti-oxidants. Anti-oxidant capacity and phenolic ingredients of flaxseed have been examined in both *in vitro* and *in vivo* models. In many examinations, it has been shown that the anti-oxidant capability of flaxseeds or their extracts is associated with their phenolic content (18). As a result of its *in vitro* anti-oxidant capacity, SDG is the most examined phenolic component of flaxseed. SDG exhibited anti-oxidant activity by either direct radical scavenging or by inhibition of lipid peroxidation (19, 20). SDG scavenges concentration-dependent produced •OH radical and also inhibited the lipid peroxidation of liver homogenate in a concentration-dependent manner. The useful flaxseed anti-oxidant components aid to restore the increased activity of hepatic enzymes at almost normal levels (21). Furthermore, in another study, it has been manifested that decreased ROS production and elevated ROS elimination result from high consumption of dietary flaxseed which increases anti-oxidant protection. Probably the anti-oxidative activity of flaxseed depends on water-soluble substances. It can be concluded that proteins are vital for the anti-oxidative activity of flaxseed. Anti-oxidative activity of proteins and their roles in activation or elevation of anti-oxidant features of components has been demonstrated (22).

***Anti-diabetic effects***

Diabetes is a metabolic disease resulting from a deficiency in insulin secretion and insulin function. Diabetes is determined by hyperglycemia, glycosuria, polyuria, polydipsia, polyphagia, devastated *glucose* tolerance, insulin resistance, hyperlipidemia, and diabetic coma (23). Diabetes is principally categorized into type 1 and type 2. It has been exhibited that biomarkers of oxidative stress are increased in both major forms of diabetes (24). Notable evidence demonstrates that hyperglycemia leads to the production of reactive oxygen species (ROS) which can result in oxidative stress and cellular damage. In hyperglycemia, glucose autoxidation and non-enzymatic glycation of proteins lead to elevation of mitochondrial ROS which causes oxidative stress. Much data manifest the participation of ROS in the beginning and development of diabetes mellitus. It is accepted that vascular complication, especially in type 2 diabetes, is progressed by the crucial role of oxidative stress (25). Progression of diabetic damages depends on different levels of enzymes like *catalase *(CAT–enzymatic/non-enzymatic), superoxide dismutase (SOD), and glutathione peroxidase (GSH–Px) which make tissues vulnerable to oxidative stress. Increased hepatic gluconeogenesis is the main cause of hyperglycemia in patients with type 2 diabetes (26). 

Diabetes is controlled by flaxseed as a natural source. Anti-oxidant activity and decrease of glucose levels in serum caused by inhibition of *phosphoenolpyruvate carboxykinase *(PEPCK) gene expression are two anti-diabetic mechanisms of flaxseed. It has been presented that plasma glucose homeostasis is affected by SDG-containing foodstuff as well as flaxseed fibers. The creation of diabetes mellitus is inhibited by SDG derived from flaxseed. The amount of lignans in flaxseed is 1000 times more than other food sources such as sesame seed, pumpkin seeds, cereals (wheat), leguminous plants (lentils, soybeans), fruits (pears, prunes), and certain vegetables (garlic, asparagus, carrots), so it is considered as the main source of lignans with SDG as a basic compound. Flax lignan complex (FLC) derived from flaxseed is composed of 34% to 38% SDG, 15% to 21% cinnamic acid glucoside, and 9.6% to 11.0% hydroxymethyl glutaric acid by weight. Insulin resistance in type 2 diabetes which is related to C-reactive protein condensation and progression of diet-induced obesity was decreased by SDG (27). FLC decreased the metabolic syndrome composite score in males; however, no effects were observed in females (28). It was shown that reduction in serum and pancreatic malondialdehyde (MDA) causes decreased oxidative stress which is related to these effects. It was shown that SDG decreases serum MDA and *glycated hemoglobin* (A1C) levels which are related to delayed development of diabetes in the Zucker fatty rat type 2 diabetes model (29). It can be concluded that SDG delays type 2 diabetes in animal models and causes inhibition and delaying of development of diabetes by decreased oxidative stress and also suppression of PEPCK gene expression results in prevention and delaying of the progression of diabetes.

***Effects of flaxseed on cardiovascular diseases***

According to the World Health Organization (WHO), cardiovascular diseases (CVD) are the number 1 cause of death globally: 17.9 million people die each year from CVDs. One-third of these statistics are for countries with below-average incomes. A healthy diet and normal body mass index (BMI) can reduce the risk of CVDs. Hypertension*,* hyperlipidemia, atherosclerosis, and platelet aggregation (30) can be managed by herbal supplements like flaxseed. The most important bioactive components of flaxseed, which have a positive effect on cardiovascular risk factors and CVDs, include *α-linolenic acid* (ALA), ω-3 (n-3) *polyunsaturated fatty acids* (PUFAs), lignan phytoestrogen, SDG, and proteins (31). 

Many animal and clinical studies have shown the antihypertensive effects of flaxseed. Some interesting research in this field is given below. The prospective, double-blinded, placebo-controlled, randomized trial reported 30 g/day of milled flaxseed for 6 months could reduce blood pressure in patients (32). Flaxseed proteins and peptides, which are rich in *arginine* residues, display an antihypertensive effect in rats, and also flaxseed fiber in doses 7.2 to 18.9 significantly decreased blood pressure. The majority of animal and clinical studies suggested that consumption of flaxseed products leads to a decrease in serum lipid profile such as total cholesterol (TC), LDL-cholesterol, triglyceride (TG), lipoprotein a (Lp a), Serum apolipoprotein A-1, and apolipoprotein B (33). However, there are conflicting articles against these results, which can be interpreted by animal species, study model, the dose of flaxseed, duration of consumption, and the target studied patients. Flaxseed in animal studies has been shown to reduce arrhythmias, reduce the size of the infarct, reduce apoptosis and inflammatory factors, and enhance the anti-oxidant capacity in the heart (34, 35). Flaxseed can affect the cardiovascular system by the following mechanisms:

1) Flaxseed has a powerful anti-oxidant activity and also it can decrease caloric intake. 

2) flaxseed SDG increases the neovascularization in the heart and thereby improves vascular and cardiac function.

3) Flaxseed has shown anti-atherogenic activity, antiplatelet action, and improvement in cardiac cell survival through reducing inflammatory cytokine production, inhibition of anti-aggregatory prostacyclin, and up-regulating anti-apoptotic proteins, respectively.

***Effects of flaxseed on gastrointestinal disorders***

Gastrointestinal disorders include such conditions as colon cancer, functional gastrointestinal disorders (FGID) such as functional dyspepsia (FD), diarrhea, and irritable bowel syndrome (IBS) as well as inflammatory bowel disease (IBD). Healthy lifestyle, good bowel habits, and performing cancer screening decrease or inhibit gastrointestinal disorders (36). The effects of consuming 50 g/day flaxseed for 4 weeks on different factors of nutrition in young healthy adults were characterized by Cunnane *et al*. (1995). It was manifested that consuming flaxseed increased bowel movement by 30% per week (*P*<0.05). Fibers may also play a role through the constitution of small-chain fatty acids, e.g., acetate and propionate. It can be concluded that augmentation of bile acid expulsion rate and decrease of bile acid reabsorption may be affected by these small-chain fatty acids via increased fecal excretion of cholesterol, and increasing colonic mobility, and inciting colonic transit (37). Reciprocally, researchers examined cases with IBS in an open randomized controlled trial. There were no significant changes in stool frequency or stool consistency between control and flaxseed groups (38). 

Another theory proposes that the prevention of calcium channels shows antimotility and antisecretory activity which can cause antidiarrheal effects of flaxseed (39). Anaya *et al.* stated that antinutritional factors and low protein digestibility reduce the nutritional value of flaxseed (40). Besides this, it was presented that digestive enzyme inhibitors of flaxseed impaired rat growth and reduced intestinal villi. Bowel inflammation and functional gastrointestinal diseases are treated by the increase of intestinal bulk which results from the water-binding capacity of the insoluble fiber in ﬂaxseed (37). 

The activities of chemoprotection, anti-inflammation, and antimicrobe along with augmentation of bowel movement and releasing bile acid are the advantages of flaxseed consumption as a nutritional supplement. Regulation of expression of genes involved in cell cycle arrest and mitochondrial apoptosis, as well as K+ channel opening mechanism, are two main mechanisms that are involved in the effects of flaxseed on GID. 

***The effect of flaxseed on hepatic disorders***

Redox enzymes and bioenergetic electron transfer living organisms are involved in free radicals and reactive oxygen species (ROS) production from exogenous or endogenous sources. Oxidative stress mediated by free radicals and ROS results in proteins, lipids, and nucleic acid disruption which cause disorders including cancer, diabetes, atherosclerosis, and hepatic diseases. Anti-oxidant compounds play a preventive role in dealing with these molecules (41). Several animal and human models confirmed the anti-oxidant properties of SDG and its metabolites. Reduced levels of total protein and albumin are observed in rabbit induced hepatotoxicity model as compared with the control group. A remarkable supportive effect for SDG is proposed in the liver and renal complications caused by paracetamol-induced hepatonephrotoxicity in rabbits. Accordingly, flaxseed lignan therapeutically is rendered a potential hepato-nephroprotective representative (42). Furthermore, flaxseed supplied appropriate therapeutic management to control hypertriglyceridemia and fatty liver in rats. Another study indicated SDG lignans histological protective effects on non-alcoholic fatty liver disease (NAFLD) in rabbits. Also, reduced liver disease risk factors in hypercholesterolemia are suggested as flaxseed lignan preventive properties. Besides, blood cholesterol decreased following oral administration of SDG in human hypercholesterolemia (43). Collectively, numerous studies showed that flaxseed oil as a rich source of n-3 fatty acids alleviates oxidative stress and lipid accumulation in the liver, suppresses hepatic glycolytic and lipogenic genes, improves insulin sensitivity and reduces fat absorption based on a large amount of lignan and soluble fiber leading to hypolipidemia (44). It must be mentioned that the reduction in the mRNA expression levels of sterol regulatory element binding protein-1c (SREBP-1c), which regulates the activity of cholesterol and fatty acid synthetase enzymes, can lead to enhanced blood cholesterol-lowering effect and reduce the risk factors of liver disease.

***Anticancer effects of flaxseed***

Improper signaling in biological pathways associated with cell proliferation, inflammation, and oxidative stress, among others cause cancer and other chronic diseases. It is demonstrated that plant foods contain phytochemicals or bioactive compounds such as lignans that reduce cancer risk or progression. Some foods such as fruits, vegetables, seeds, the bran layer of grains, and legumes contain phenolic compounds known as lignans. Today, as a result of the useful health effects of plant lignans such as antitumor, anti-oxidant, both estrogenic, anti-estrogenic activity, and decrease of the risk of developing chronic diseases, especially cancer, they are becoming a great therapeutically active class of compounds. SDG can be converted to mammalian lignans *enterodiol *and *enterolactone* by bacteria in the animal or human colon. Flaxseed is considered a great anticancer food because of its significant level of SDG and ALA (45). It has been demonstrated that strong antiproliferative, anti-oxidant, antiestrogenic, and/or anti-angiogenic activities of SDG prevent some malignant tumors such as breast, lung, and colon cancer. The inhibition of enzymes involved in carcinogenesis is the suggested mechanism for the anticancer effect of SDG (43). It has been demonstrated that consuming SDG reduces the volume, area, and number of tumors. The primary risk factor for colon cancer is characterized by the growth of crypts and crypts foci. In several studies, it has been manifested that aberrant crypts and their foci show the anticancer role of flaxseed SDG and lignan supplementation in the diet (46). Colon tumor cell proliferation may be prevented by lignans through apoptosis-mediated cell death and also through their anti-oxidant feature. The circulating level of ALA, EPA, and DHA which were caused by consumption of dietary flaxseed, could be effective for the management of colon cancer. It has been exhibited that dietary phytoestrogens prohibit and treat breast cancer and also result in both weak estrogenic and anti-estrogenic activities (47). It has been shown that α-Linolenic acid has anti-inflammatory effects, and also antiproliferative activities were shown in premenopausal animal models of breast cancer. Angiogenesis is considered a basic step in cancer development. Vascular endothelial growth factor (VEGF) is one of the main stimulants of angiogenesis (48). Already it has been proven that extracellular VEGF increases by estradiol (E2) in cancerous and normal human breast tissue *in vivo*. In a study, it has been presented that estrogen receptor-positive breast cancer may be prevented by the anti-estrogenic effects of flaxseed and its lignans (49). Breast cancer is prevented by SDG through modification of the expression level of zinc transporters since the zinc levels in breast cancer cells are more than normal ones (50). In a study on 14 healthy men, it was presented that diets of 50 to 60 g of fiber from mixed sources decrease prostate-specific antigen (PSA) (51). Researchers demonstrated that the risk of prostate cancer is directly associated with prostatic fluid lignin levels (52). Flaxseed contains many dietary lignans. It has been demonstrated that total and free testosterone and 5α-reductase (testosterone converter enzyme to dihydrotestosterone) are decreased by lignans. In a hormone-related neoplasm such as prostate cancer, these effects may be important (53). Flaxseed is a rich source of plant-based N-3 fatty acids (N3FA), which have been shown to play important roles like increasing the natural killer cell activity, changing the tyrosine kinase cell signaling pathways, preventing the cell membrane synthesis, influence cell receptor status, and impact the eicosanoid milieu (suppressed production of prostaglandins E2 and 5-hydroxyeicosatetraenoic acid) via cyclooxygenase and lipoxygenase pathways (53).

It can be concluded that some of the compounds, such as lignans (through antitumor, anti-oxidant, both estrogenic, anti-estrogenic activity), modulate the signaling mechanism of cell proliferation, inflammation, and oxidative stress and thereby can reduce the risk of cancer or its progression.

***The effect of flaxseed on urological disorders***

The kidney is the main target organ for noxious components (54). Throughout the world, kidney failures are considered an important health problem. Also, the percentage of deaths increases due to renal dysfunctions yearly (55). Different reasons such as chemical toxicity, sepsis, and trauma cause acute renal damage. Globally, medical herbs play an important role in the treatment of several illnesses (56). Exacerbation of renal injury and development of chronic renal failure is directly associated with high protein consumption. Moreover, renal function and renal disease may be improved by changing the source or type of dietary protein (57). Plant seeds, especially flaxseed, play an important role in the treatment of chronic renal disease and human lupus nephritis. It was demonstrated that flaxseed consumption instead of dietary protein decrease proteinuria and glomerular and tubulointerstitial lesions in obese SHR/N-cp rats and also flaxseed meal decreases proteinuria and renal histologic abnormalities more effectively, compared with soybeans (58). Evidence showed that mortality, proteinuria, and decline in glomerular filtration rate (GFR) are reduced by flaxseed diet (59).

In several studies, it was exhibited that thioacetamide-induced renal injury is prevented by flaxseed oil and its anti-oxidant activity causes the protective effect (60). Severe renal injuries among older adults who are dependent on dialysis or transplantation for survival can be created by chronic kidney disease (CKD). Both flaxseed oil either used as a food or as a curative supplement and whole flaxseed may have similar effects on the treatment of renal injury (61).

In a study, nephroprotective effect of ethanolic extract of flaxseed/*L. Usitatissimum* (EELU) in high dose in gentamycin-induced nephrotoxicity model in Wistar rats and its anti-oxidant potential in the prevention of acute kidney injury was introduced. Acute kidney injury is primarily created due to renal ischemia-reperfusion (IR) (62). Disorders in Cellular Ca^2+^ metabolism, high levels of free radicals, and generation of toxic lipid metabolites were suggested as involved mechanisms in this respect. IR-induced acute kidney injury can be prevented by supplementations with omega-3 fatty acids, particularly eicosapentaenoic acid (EPA) and docosahexaenoic acid (DHA), which are the principal (n-3) polyunsaturated fatty acids (PUFA) found in fish. The results demonstrate the beneficial effects of flaxseed and fish oil in the reduction of IR-induced renal injury. It was manifested that histological and oxidative stress damages were decreased by 4-week treatment with flaxseed oil and fish oil (63).

***The effect of flaxseed on reproductive disorders***

The valuable effects of lignan complex and alpha-linolenic acid from flaxseed on reproductive disorders have been investigated (64). It was shown that early malnutrition-associated changes are reversed by flaxseed flour (contained 17% protein, 45% carbohydrate, 26% fat, and fibers) or oil (contained 13% linoleic acid and 52% α-linolenic acid). 

*Polycystic ovary syndrome* (PCOS) is a female hormone-related disorder. Hormonal imbalance in PCOS often causes infertility problems in women. Androgen levels of plasma mostly increase in PCOS patients. Elevated production of androgens in PCOS patients can be caused by activation of the hypothalamic-pituitary-adrenal (HPA) axis due to metabolic disorders such as insulin resistance. Changing of lifestyle, surgery, and medication such as *clomiphene citrate, metformin, letrozole, and tamoxifen* have been suggested as curative methods for PCOS (65). Today, herbal medicines have become a popular method in the treatment or control of the disease. In a case study, it was manifested that flaxseed consumption (30 g/day for 4 months) decreased testosterone and increased the insulin level in a 31-years old woman with PCOS (66). In many studies, it was reported that flaxseed lignans decrease the excess testosterone which is responsible for PCOS pathogenesis. Any substances that can decrease androgen levels will be beneficial in androgen-dependent disorders such as PCOS. In a study, it was demonstrated that flaxseed consumption not only decreases the ovarian volume and number of follicles in polycystic ovaries but also regulates the rate of menstrual cycles without any effects on body weight, blood sugar, and hirsutism. Improvement of follicular maturation and the anti-inflammatory effect to decrease ovarian volume result from the effect of FSP by the reduction in testosterone, estrogen, LH, and insulin levels. As a result of beneficial effects on ovarian function and menstrual cycle, flaxseed is suggested to be a drug source for PCOS treatment (67).

As a result of reduction in the circulating levels of estrogen, menopausal women are more susceptible to cardiovascular disease (CVD). Disorders of menopause are related to a more atherogenic lipid profile and changed vascular responsiveness. It has been demonstrated that endothelial activity which plays an important role in development, progression, and clinical manifestations of atherosclerosis and aorta distensibility decrease around the time of menopause (68).

Disease prevention should occur during life, beginning with the mother’s diet during gestation, throughout lactation, and into adulthood (69). In several studies, it has been manifested that prenatal and primary postnatal nutrition affect the susceptibility to chronic diseases related to obesity, hypertension, and cardiovascular diseases in adulthood (70). As a result of the beneficial effects of functional foods such as flaxseed in the prevention of diseases as well as their role in losing weight and decreasing body mass index (BMI) they have been studied frequently (71).

**Table 1:** Biological activities of flaxseed against different disorders based on experimental studies

Authors (year)	Disorder	Target	Effective dose	Main results
Kailash Prasad (2000) (72)	**diabetes**	**rats**	22 mg/kg body weight SDG, orally	Decreasing serum and pancreatic MDA and WBC Increasing pancreatic antioxidant reserve
Noureddin Soltanian (2018) (73)	**diabetes**	**human**	10 g of flaxseed pre-mixed in cookies	Decreasing constipation symptoms, weight, glycemic, and lipid levels
Pei Hua (2015) (74)	**diabetes**	**mice**	(3, 10, or 30 mg/kg)	Analgesic actions of SDG in diabetic mice may be associated with its antioxidant activity
Barre ***et al.*** (2012) (75)	**diabetes**	**human**	600 mg total SDG/day	Increasing bleeding time Reducing the prothrombotic state reduced central obesity gain
Muslum Gok (2015) (76)	**diabetes**	**rats**	0.714 g/kg body weight/day; orally flaxseed	Modulating G6PD, 6PGD, GR, and GST activities in tissues
Hadjighassem ***et al.*** (2015) (77)	**healthy people**	**human**	500 mg of alpha linolenic acid	Plasma levels of BDNF and MDA significantly increased.
Saad ***et al.*** (2014) (78)	**non-alcoholic fatty liver disease**	**rabbit**	8 mg/kg of ground flaxseed	Reduction of triglyceride levels.
Xu ***et al.*** (2013) (44)	**non-alcoholic fatty liver disease**	**rats**	8 g/kg	Elevated hepatic antioxidant defense capacities.
Rodriguez ***et al.*** (2013) (79)	**peripheral artery disease**	**human**	30 g/d for 6 months	Anti-hypertension
Edel ***et al.*** (2015) (80)	**CVD**	**human**	30 g for 12 months	Reduction of TC and LDL cholesterol
Bloedon ***et al.*** (2008) (81)	**hypercholesterolemic adults**	**human**	40 g/day for 10 weeks	Reduction of Lipoprotein A Improves insulin sensitivity.
Ghule ***et al.*** (2015) (34)	**cardiac hypertrophy rat model**	**rats**	400 mg/kg per day for 4 weeks	Improves VEGF Reduction of TNFα Improvement of cardiac function.
Hernández-Salazar ***et al.*** (2013) (82)	**colon cancer**	**rats**	16 g/d flaxseed for 10 weeks	Reduction of Crypt multiplicity
Palla ***et al.*** (2015) (39)	**diarrhea **	**mice**	100, 300, and 500 mg/kg	Anti-microbial
Millman ***et al.*** (2019) (83)	**healthy mice**	**mice**	35% for 10 weeks	Improves anti-microbial peptide
Rizwan ***et al.*** (2014) (84)	**renal toxicity**	**rats**	15% flaxseed oil by weight to normal diet	Attenuated the NaAs-induced changes
Moghimian (2019) (63)	**IR injury**	**rats**	0.4 g/kg	Protection against IR-induced renal injury
Sankaran ***et al.*** (2007) (85)	**CKD**	**rats**	7% flax oil in diet	Antioxidant effects Anti-inflammatory effects
Jelodar ***et al.*** (2018) (86)	**PCOS**	**rats**	200 mg/kg hydroalcoholic extract of flaxseed	Sex-steroid hormonal profile was ameliorated
Lucas ***et al.*** (2002) (33)	**menopausal disorder**	**human**	40 g/day of flaxseed	Improves lipid profiles No effect on biomarkers of bone metabolism

SDG: secoisolariciresinol diglucoside; MDA: malondialdehyde; WBC-CL: white blood cell; G6PD: glucose-6-phosphate dehydrogenase; 6PGD: 6-phosphogluconate dehydrogenase; GR: glucocorticoid receptor; GST: glutathione S-transferase; LDL: low-density lipoprotein; CVD: cardiovascular disease; TC: total cholesterol; TG: triglyceride; IBS: irritable bowel syndrome; IR: renal ischemia-reperfusion; CKD: chronic kidney disease; PCOs: polycystic ovary syndrome; VEGF: vascular endothelial growth factor; TNFα: tumor necrosis factor α; NaAs: sodium arsenite; pH: potential hydrogen

## Conclusion

Flaxseed has multiple compounds with bioactive plant materials such as oils, lignans, vitamin E, dietary fibers, and potassium and is full of anti-oxidant activity and by this activity, reduces the risk of cancer, diabetes, and renal injury and also prevents malignant tumors in the breast, lungs, and colon. Direct radical scavenging and inhibition of lipid peroxidation are the two major mechanisms of the anti-oxidant activity of flaxseed. The peptides and potassium of flaxseed reduce the risk of blood platelet aggregation, blood pressure, and free radicals in the blood and finally decrease the risk of cardiovascular disease. Due to its role in increasing the excretion of sodium in urine, Vitamin E is an important component of flaxseed which leads to a decrease in blood pressure and heart disease. Prevention of calcium channels shows antimotility and antisecretory activity which can cause antidiarrheal effects of flaxseed. Flaxseed oil decreases the accumulation of lipids, suppresses hepatic glycolytic and lipogenic genes, improves insulin sensitivity, and reduces fat absorption. Based on the information and mechanisms mentioned above, flaxseed is recognized as a medical plant. Future studies may challenge what we now believe about the therapeutic effects of flaxseed or confirm the mentioned findings. We believe that more clinical studies are needed to present a complete list of flaxseed effects.
